# Inequality in time to first antenatal care visits and its predictors among pregnant women in India: an evidence from national family health survey

**DOI:** 10.1038/s41598-023-31902-3

**Published:** 2023-03-22

**Authors:** Abhipsa Tripathy, Prem Shankar Mishra

**Affiliations:** 1 Department of Mathematics and Computing, Indian Institute of Technology (ISM), Dhanbad, Jharkhand 826004 India; 2 Department of Population Research Centre, Institute for Social and Economic Change, Bengaluru, Karnataka 560072 India

**Keywords:** Health care, Health occupations

## Abstract

For countries with high maternal mortality and morbidity, on-time initiation of antenatal care (ANC) is indispensable. Therefore this paper aims for studying the median survival time (MST) of first ANC among pregnant women as well as understanding the contextual factors that influence a mother’s decision to access ANC services in India. The study used cross-sectional survey data obtained from the NFHS-4 conducted in 2015–2016. The MST of the timing of the first ANC visit was estimated using the Kaplan-Meir estimate. A multivariate Cox-proportional hazard regression model was used to identify the factors related to the timing of the first ANC visit with a 95% confidence interval (CI). Overall at least one ANC checkup was assessed by 60.15% of women and the median survival time for the first ANC checkup was found to be 4 months. Early initiation of ANC in pregnant women increased by 37% (AHR: 1.37, CI:1.34–1.39) for primary education, and 88% (AHR:1.88, CI:1.86–1.90) for secondary education compared to women having no formal education. Results of the current study revealed that the median survival time of the first ANC visit was 4 months in India which is delayed compared to recommendations of WHO. Therefore boosting the access and utilization of antenatal care coverage among pregnant women can ensure the best health outcomes for their pregnancy.

## Introduction

Despite several policies and programs interventions at international and national levels in order to improve the mothers and children's survival, by providing comprehensive, integrated, and a continuum of care to them. However, the low coverage and under-utilization of essential care services influence mothers and infants’ survival^1^. Maternal healthcare still remains a top global and national priority and challenges continued in many low and middle-income countries^1,2^. A current global estimate shows that every day nearly 810 women died from preventable causes related to pregnancy and childbirth. At an aggregate level, 295,000 maternal deaths occurred in 2017 which has dropped considerably from 451,000 deaths in 2000^1^. Out of the total maternal deaths, 99% occur in only developing countries compared to developed countries^1,3^. India, in South Asian countries, contributes a higher share of maternal deaths. For instance, the maternal mortality ratio (MMR) for the period 2016–2018, as per the latest report of the National Sample Registration System (SRS) data is 113 per 100,000 live births^4^, which is still a way higher than the Sustainable Development Goals’ target that is 70 per 100,000 live births.

Most of maternal and child deaths and complications related to pregnancy could have been prevented through the appropriate access and use of essential life-saving maternal care services. In fact, the coverage of life-saving health interventions and practices among pregnant women remains low due to gaps in knowledge, policies, and availability of resources, and supply-side constraints in the community^5^. For instance, antenatal care (ANC) coverage is an indicator of access and use of health care during pregnancy and its appropriate use can curb down maternal and child mortality rates. Hence, ANC is defined as “the routine health control of presumed healthy pregnant women without symptoms (screening), in order to diagnose diseases or complicating obstetric conditions without symptoms, and to provide information about lifestyle, pregnancy and delivery^6^”. The antenatal period presents opportunities for reaching pregnant women with interventions that may be vital to their health and wellbeing and that of their infants. Based on the benefits of ANC, World Health Organization(WHO) recommends that pregnant women should attend at least four ANC visits to increase opportunities for risk stratification and/or the identification, prevention, and management of pregnancy and/or co-morbidities, as well as health promotion^3^.

Awareness and responsible utilization of high-quality ANC during the first trimester of pregnancy can act as a catalyst in the prevention of serious morbidity of the mother as well as their infants^3,7^. ANC is an umbrella of the medical procedures and care required during pregnancy. WHO has recommended the ANC model to all pregnant women to follow up within the first trimester of pregnancy. Further, in 2016 the WHO revised its recommendations to a minimum of eight ANC check-ups, as per the schedule the first contact takes place in the first trimester of pregnancy (up to 12 weeks of gestation) and two contacts are scheduled in the second trimester and (at 20 and 26 weeks of gestation) and five contacts scheduled in the third trimester (at 30, 34, 36, 38 and 40 weeks)^6^. Still, these patterns of ANC visits are hardly followed and implemented in India. However WHO recommendations for minimum ANC visits was limited to only four check-ups before 2016, during which the data was collected used in the study. Lack of timely initiation of ANC or no ANC can increase the risk for mother and child^8,9^. Women who are not initiating ANC early may lead to late diagnosis of complications which might have the potential to detrimentally affect maternal and foetus health^10,11^. Therefore, the utilization of quality and timely ANC is an essential element of efforts. Yet, many women missed and compromised the first ANC visits that could have minimised pregnancy-related complications. At the country level, the percentage of women who had received ANC in the first trimester was around sixty percent, which was reduced to about fifty percent for at least 4 ANC visits and the percentage is even less for mothers who had full ANC checkups (21 percent)^12^. Even though it is also seen a high inequity across different stratum of the community and regions.

Although there are various kinds of literature studying different factors that influence the timing of the first ANC visit among women^8–10^, however, there is a dearth of literature primarily focusing on the exact time for first ANC visit in Indian context. Therefore, studying the median survival time (MST) as well as understanding the contextual factors that influence a mother’s decision to attend, is of utmost importance. It also plays a crucial role in policy making for healthcare practitioners and policymakers, as it can offer relevant information and opportunities for targeted policy interventions. Appropriate utilization of first ANC among pregnant women has paramount importance for enhancing newborn and maternal life and reducing significantly morbidity and mortality among them^6^. Therefore this paper aims to understand the timing of the first ANC visit and its predictors among pregnant women in India.

## Materials and methods

### Study design, area and period

The study used cross-sectional survey data obtained from the National Family Health Survey round 4 (NFHS-4) conducted in 2015–2016, and the information to the respondent was collected for five years preceding the survey^12^. The survey collects information on reproductive, maternal and child health care services. The detailed socio-demographic and household characteristics were provided by women respondents. The survey was carried out throughout India, covered all 29 states, and seven union territories, and for the first time gave estimates for 640 districts in India^12^.

### Sample Size determination and sampling techniques

NFHS-4 adopted a two-stage stratified sampling design for the selection of the sample. In all, 28,586 Primary Sampling Units (PSUs) were selected across the country in NFHS-4, of which fieldwork was completed in 28,522 clusters^12^. The 2011 census served as the sampling frame for the selection of PSUs. PSUs were villages in rural areas and Census Enumeration Blocks (CEBs) in urban areas. Within each rural stratum, villages were selected from the sampling frame with probability proportional to size (PPS). The final sample PSUs were selected with PPS sampling. In urban areas, CEB information was obtained from the Office of the Registrar General and Census Commissioner. CEBs were sorted, and sample CEBs were selected with PPS sampling. Selected PSUs with an estimated number of at least 300 households were segmented into segments of approximately 100–150 households^12^. Two of the segments were randomly selected for the survey using systematic sampling with probability proportional to segment size. Therefore, an NFHS-4 cluster is either a PSU or a segment of a PSU. In the second stage, in every selected rural and urban cluster, 22 households were randomly selected with systematic sampling. Further details on the data source can be ascertained from the report^12^.

#### Study participants

**Figure 1:**
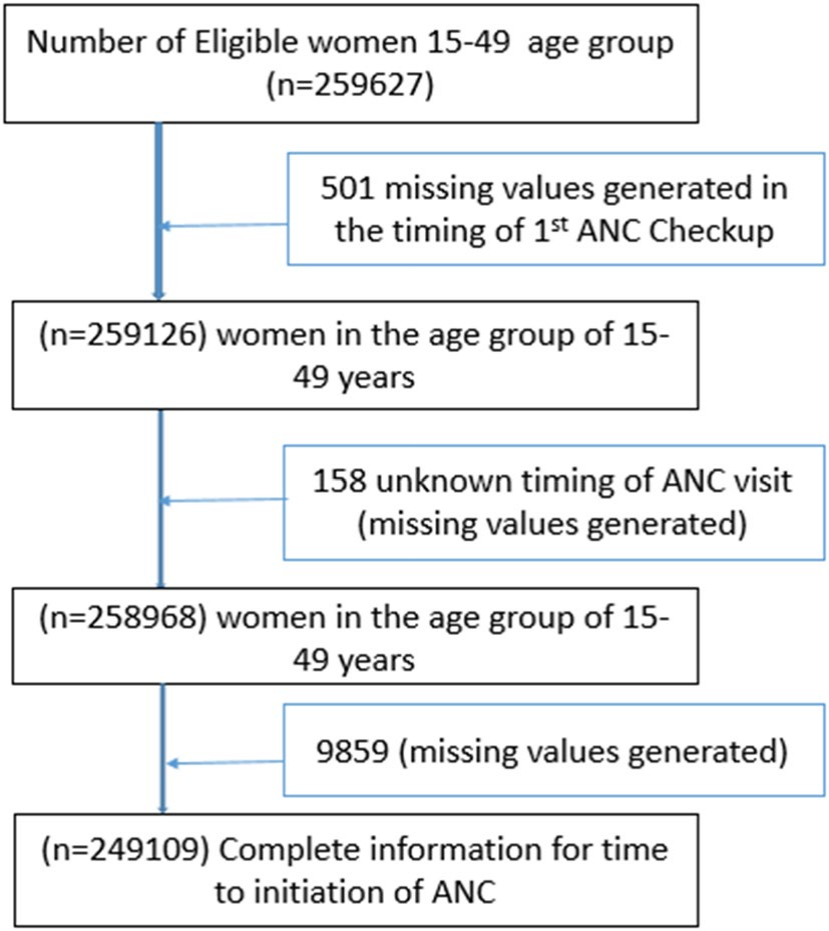
Flow chart showing sample size and sampling procedure to reach the final sample size.

The study is particularly focused in the timing of the first ANC visit to its beneficiaries^9,10^. It included all pregnant women in the age group of 15–49 years. The survey collected information from a total of 601,509 households with a response rate of 98 percent. A total of 699,686 women aged 15–49 years and 112,122 men aged 15–54 years with a response of 97 per cent and 92 per cent, respectively, were interviewed. The present study used the kid’s data file for the analysis (n = 259,627). The analysis of the study is controlled for 249,109 samples (Fig. 1). In Kid’s data set, the information collected for children aged 0–59 months as well as their mothers’ information regarding reproductive, maternal and child health and nutrition. Furthermore, child’s immunisation, nutrition, health status, disease prevalence morbidity and mortality related aspects are provided in the dataset.

### Variables under study

The dependent variable in the current study is the time to the first ANC visit which is estimated in months. The time variable in the study is the time period from pregnancy to the first ANC visit. Time component was taken as the time for the first ANC in a month(s) if the women had followed up further, otherwise gestational period for the corresponding pregnancy was taken into consideration. During pregnancy, gestational age is frequently used to express how far along the pregnancy is. A typical pregnancy can last between 38 and 42 weeks. Infants born before 37 weeks are considered premature. Infants born after 42 weeks are considered post mature. As a result, the average gestational period is 36 weeks or 9 months as considered in the study. The status of the event of interest is considered 1 (status = 1) if a woman opted for an ANC check-up and 0 (status = 0) otherwise. All the events with status 0 are known as censored in survival terms. The independent variables were chosen based on a literature review that included some socio-demographic factors known to influence the timing of ANC^8–11^. These variables are age, level of education of women, residence, religion, caste, wealth index, mass media exposure, birth order and head of the household, etc. The age variable is categorised into three categories “15–24”, “25–34”, “35–49” and education into four categories such as “no education”, “primary”, “secondary”, and “higher”. Similarly, the variable religion is categorised into four categories like “Hindu”, “Muslim”, “Christian” and “others” while caste is into four categories as “Scheduled Castes (SCs)”, “Scheduled Tribes (STs)”, “Other Backward Classes (OBCs)” and “Others”. The nominal variable mass media exposure is divided into two categories like “yes” and “no”. The ordinal variable wealth quintile index is originally classified into five groups such as “poorest”, “poorer”, “middle”, “richer”, “richest”, which we further classified into three groups as “poor”, “middle” and “rich” for ease of analysis. “Poorest”, “poorer” are clubbed into “poor”, “middle” remained as “middle” and “richer” and “richest” are clubbed into “rich” category. Obstetric factors such as birth order is grouped into four groups “1”, “2”, “3” and “4 + ”.

### Statistical survival analysis

In the present study, pregnant women are the population at risk since they are exposed to the “risk” of visiting healthcare facilities for ANC services during their pregnancy. We are evaluating the survival function S(t) and hazard function h(t) of the timing of the first ANC visit in this study, technical definitions of which are described below. Survival probability is defined as the probability of pregnant women not accessing ANC service within the specified time t. It is the probability of not failing before time t, however, the hazard function gives the instantaneous probability of failing after time t, given that the event has not occurred before time t. Here “failing” means up-taking the ANC service^9,10^.

#### Survival time

Survival time was measured in month(s) from time of pregnancy to time for first ANC visit for all the women having at least one ANC visit, otherwise their current gestational age was considered.

#### Status

The event under consideration (ANC visit) was said to happen if the pregnant woman had at least one ANC visit and its status was marked 1, otherwise it was considered as a censored event with status 0.

#### Region

Total number of states in India has been divided in three groups based on their performance on socio-economic and health indicators. Poor-performing Empowered Action Group (EAG) states are grouped together with Assam as “EAG + Assam”, while relatively high-performing states are categorised as “Southern” states and the rest other states are considered as “Others”.

For this study, secondary data have been used and analysed using R studio software. After extracting and cleaning the data descriptive measure like a frequency table is used to characterize the study population with respect to different predictor variables. Median survival time was estimated for the timing of the first ANC visit using Kaplan–Meier survival curve^10^. Survival time is given by,

$${\text{S}}\left( {\text{t}} \right) = {\text{Pr}}\left( {{\text{T }} > {\text{ t}}} \right) = {1} - {\text{F}}\left( {\text{t}} \right)$$

where T is a random variable representing time until the event of interest, and F(t) is the cumulative distribution function. In censored survival data, median survival time reflects the period before half of the individuals experience the event of interest. It is the number of months before half of the women went ahead for ANC services. While comparing two groups of their survival experiences, only the survival curves are not sufficient in order to conclude that they have significant differences in their survival. The curves may have greater difference at some point of time and it may vary eventually. Hence the Log-rank test was used to assess the difference in survival time for ANC between groups of categorical variables with the desired outcome which takes the whole follow-up time into account. The null hypothesis used in this test is that there lies no difference between the groups in the probability of an event at any point of time. Here the event indicates time taken for first ANC visit. Multivariate Cox-proportional hazard (Cox PH) model compares the survival between two or more groups with simultaneous adjustment of confounding due to one or more covariates^9,10^. The hazard ratio is the estimate of the hazard rate between two groups namely the control and treatment groups. If HR > 1, the particular group is experiencing higher rate of ANC events than that of the reference group. Similarly, if HR < 1, the group is less susceptible for ANC events compared to the reference group, and if HR = 1, it implies no difference in events in the groups. To further establish the relevance of HRs, we have established a 95% confidence interval around them^8,10^.

### Ethics approval and consent to participate

The data is freely available in public domain and survey agencies that conducted the field survey for the data collection have collected a prior consent from the respondent. Local ethics committee of International Institute for Population Sciences (IIPS), Mumbai, ruled that no formal ethics approval was required to carry out research from this data source.

## Results

### Socio-demographic characteristics of the study population

Out of 249,109 study participants, around 60% (149,863) of women opted for ANC services. ANC checkup percentage is slightly less in the EAG region given in Table 1. Almost 30% (142,569) women in the study were from the age group 15–24 and around 60% (142,569) women belong to the age group 25–34 at the national level. A very few percentages of women in the age group 35–49 opt for ANC services as per the given dataset. Around one-third (31.33%) of the women in the study had no formal education, only 15% (36,563) women had primary education and the highest 45% (111,335) women had secondary education in the national level. Especially in the EAG region situation is more grieve where a larger proportion of women had no formal education (39.94%), and a fewer percentage had higher education. Of all the participants in the study around three-quarters (76.26%) were rural dwellers in the national level. The majority of the respondents belong to the poor wealth quintile in EAG states (61.50%), whereas among southern states the situation is in reverse as less than one-fifth of the respondents were in the poor wealth quintile. At the community level, most women had no exposure to mass media. Less than 40% of women (92,084) with first birth order had the opportunity for ANC service both in the national level and in EAG states whereas it is slightly high for southern states.

**Table 1:** Proportion of study participants and its distribution by demographic characteristics across regions (EAG plus Assam, Others states and Southern states) from NFHS-4 (2015–2016).

Variables	Categories	EAG + Assam n (%)	Others n(%)	Southern n(%)	Total n(%)
First ANC	Yes	86,341(56.19)	16,406(67.89)	47,116(66.11)	149,863(60.16)
	No	67,331(43.81)	7760(32.11)	24,155(33.89)	99,246(39.84)
Age	15–24	51,446(33.48)	8832(36.55)	21,350(29.96)	81,628(32.77)
	25–34	87,187(56.74)	14,093(58.32)	41,289(57.93)	142,569(57.23)
	35–49	15,039(9.79)	1241(5.14)	8632(12.11)	24,912(10.0)
Education	No education	61,380(39.94)	12,949(18.17)	3709(15.35)	78,038(31.33)
	Primary	23,765(15.46)	10,274(14.42)	2524(10.44)	36,563(14.68)
	Secondary	57,052(37.13)	40,513(56.84)	13,770(56.98)	111,335(44.69)
	Higher	11,475(7.47)	7535(10.57)	4163(17.23)	23,173(9.30)
Residence	Rural	123,999(80.69)	49,283(69.15)	16,688(69.06)	189,970(76.26)
	Urban	29,673(19.31)	21,988(30.85)	7478(30.94)	59,139(23.74)
Religion	Hindu	128,167(83.40)	35,781(50.20)	20,826(86.18)	184,774(74.17)
	Muslim	21,929(14.27)	9643(13.53)	2165(8.96)	33,737(13.54)
	Christian	1720(1.12)	18,230(25.58)	676(2.80)	20,626(8.28)
	Others	1856(1.21)	7617(10.69)	499(2.06)	9972(4.0)
Caste	Other backward classes	73,065(47.55)	16,217(22.75)	12,311(50.94)	101,593(40.78)
	Scheduled caste	31,320(20.38)	11,025(15.47)	6629(27.43)	48,974(19.66)
	Scheduled tribe	23,696(15.42)	27,251(38.24)	1005(4.16)	51,952(20.86)
	Others	25,591(16.65)	16,778(23.54)	4221(17.47)	46,590(18.70)
Wealth index	Poor	94,506(61.50)	26,133(36.67)	4341(17.96)	124,980(50.17)
	Middle	25,690(16.72)	17,276(24.24)	6455(26.71)	49,421(19.84)
	Rich	33,476(21.78)	27,862(39.09)	13,370(55.33)	74,708(29.99)
Media expose	Yes	2879(1.87)	3076(4.32)	1213(5.02)	7168(2.88)
	No	150,793(98.13)	68,195(98.68)	22,953(94.98)	241,941(97.12)
Birth order	1	52,829(34.38)	28,435(39.90)	10,820(44.77)	92,084(36.97)
	2	44,868(29.20)	22,562(31.66)	9016(37.31)	76,446(30.69)
	3	26,396(17.18)	10,648(14.94)	2886(11.94)	39,930(16.03)
	4 and above	29,579(19.25)	9626(13.51)	1444(5.98)	40,649(16.32)
Head of household	Female	18,941(12.33)	8487(11.91)	2787(11.53)	30,215(12.13)
	Male	134,731(87.67)	62,784(88.09)	21,379(88.74)	218,894(87.87)

### Time to first ANC checkup among pregnant women

**Figure 2:**
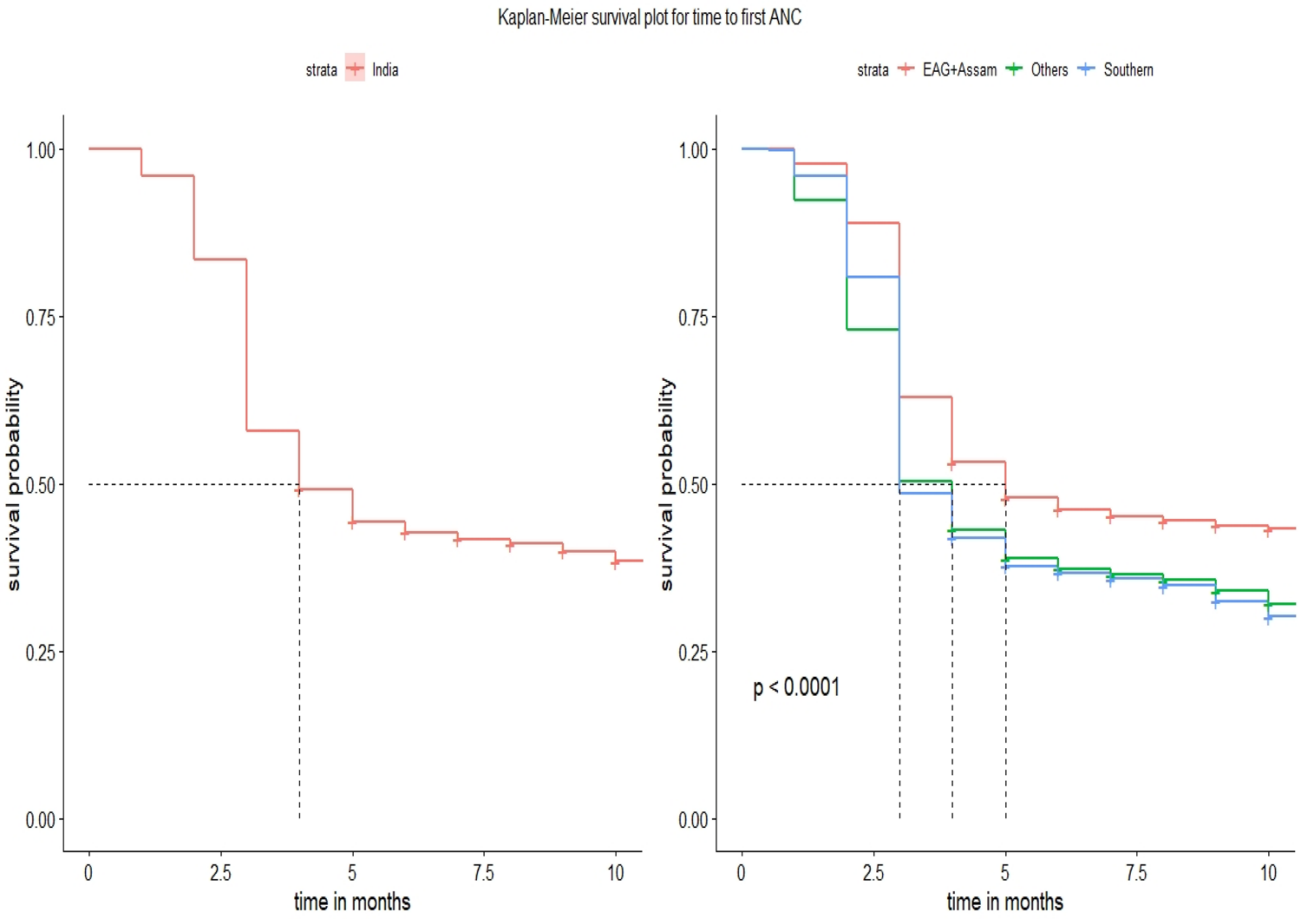
Kaplan–Meier survival estimate of pregnant women for time to first ANC visit, India and its specific regions, 2016. (*Source: NFHS-4, 2015–2016*).

The pregnant women in the current study were evaluated retrospectively for 1,383,278 person-months. Overall at least one ANC checkup was assessed by 60.15% of women and the median survival time for the first ANC checkup was found to be 4 months as in Table 2. Around one-quarter of pregnant women booked ANC services within the first trimester at the national level (Fig. 2).

**Table 2:** Proportion of study participants by background characteristics, ANC utilization, Median Survival Time, and log-rank test for timing of first ANC from NFHS-4 (2015–2016). (*Source: NFHS-4, 2015–2016*).

Variables	Category	ANC visit		MST (in months) ± IQR	Log rank test Chi-square	*p* Value
		No.	Yes			
Age	15–24	31,327(38.37)	50,301(61.62)	4 ± (6,-)	89.7	< 0.05
	25–34	57,531(40.35)	85,038(59.64)	4 ± (6,-)		
	35–49	10,388(41.69)	14,524(58.31)	5 ± (6,-)		
Education	No education	42,326(54.23)	35,712(45.76)	9 ± (6,-)	16,813	< 0.05
	Primary	15,841(43.32)	20,722(56.67)	5 ± (6,-)		
	Secondary	36,289(32.59)	75,046(67.40)	4 ± (5,-)		
	Higher	4790(20.67)	18,383(79.32)	3 ± (4,-)		
Residence	Rural	81,508(42.90)	108,462(57.09)	5 ± (6,-)	4825	< 0.05
	Urban	17,738(29.99)	41,401(70.00)	3 ± (7,-)		
Religion	Hindu	71,709(38.80)	113,065(61.19)	4 ± (6,-)	763	< 0.05
	Muslim	14,838(43.98)	18,899(56.01)	5 ± (6,-)		
	Christian	9418(45.66)	11,208(54.33)	6 ± (3,-)		
	Others	3281(32.90)	6691(67.09)	–		
Caste	Other backward classes	40,725(40.08)	60,868(59.91)	4 ± (3,-)	2288	< 0.05
	Scheduled caste	20,481(41.82)	28,493(58.17)	5 ± (3,-)		
	Scheduled tribe	22,966(44.20)	28,986(55.79)	5 ± (2,-)		
	Others	15,074(32.35)	31,516(67.64)	–		
Wealth index	Poor	62,191(49.76)	62,789(50.23)	9 ± (6,-)	16,148	< 0.05
	Middle	17,651(35.71)	31,770(64.28)	4 ± (6,-)		
	Rich	19,404(25.97)	55,304(74.02)	3 ± (6,-)		
Media expose	Yes	1842(25.69)	5326(74.30)	3 ± (7,-)	1079	< 0.05
	No	97,404(40.25)	144,537(59.74)	4 ± (6,-)		
Birth order	1	39,706(43.11)	52,378(56.88)	5 ± (6,-)	3012	< 0.05
	2	25,386(33.20)	51,060(66.79)	4 ± (2,)		
	3	15,022(37.62)	24,908(62.37)	4 ± (2,)		
	4 and above	19,132(47.06)	21,517(52.93)	–		
Head of household	Female	12,365(40.92)	17,850(59.07)	5 ± (6,-)	9.6	0.0
	Male	86,881(39.69)	132,013(60.30)	4 ± (6,-)		
India Region	EAG + Assam	67,331(43.81)	86,341(56.18)	5 ± (6,-)	4575	< 0.05
	Southern	7760(32.11)	16,406(67.88)	3 ± (6,-)		
	Others	24,155(33.89)	47,116(66.10)	4 ± (7,-)		
Total	99,246(39.84)	149,863(60.15)	4 ± (6,-)			

IQR: Inter-quartile range, EAG: empowered action group, Others region: this includes-northeastern states except- Assam, Union territories, and small states of India.

**Figure 3:**
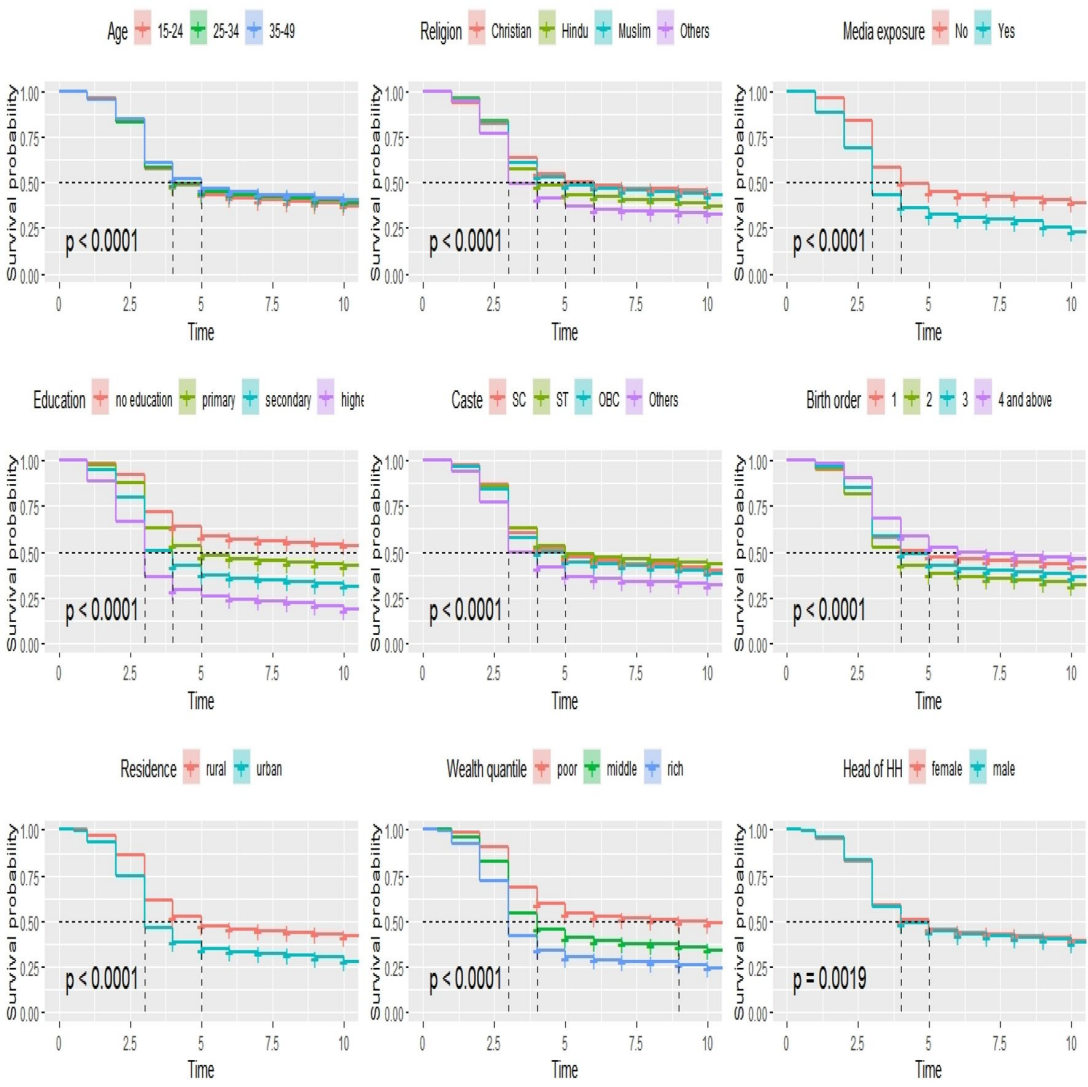
Survival estimate of time to first ANC visit by woman’s characteristics in India. (*Source: NFHS-4, 2015–2016*).

Among the women’s age, the highest ANC visits was seen by women in the age group of 15–24 years. Similarly by higher educated women (79%), women residing in urban areas (70%), women belonged to rich quintile families (74%), and those who have exposure to mass media (74.30%). The EAG plus Assam states shows the lowest utilisation in ANC visits as compared to southern and others states (Table 2). Furthermore, it also represents the median survival time for ANC checkups among women by background characteristics. Among women in the age group of 15–24 years, first antenatal care was received after 4 months whereas for women aged 35–49 first ANC was taken in 5 months. The median survival time for antenatal checkups for women with higher educational qualifications was as early as 3 months, whereas for women with no formal education it was as late as 9 months. Women residing in urban areas had their checkups within 3 months, on the contrary in the rural regions it was found to be 5 months. The first antenatal care was within the first trimester for mothers with richer wealth quintile and it was late as at 9 months for poorer wealth index. For pregnant women who got exposed to mass media, their ANC checkup time was within 3 months. Women with first and second birth orders availed ANC services within 5 months and 4 months respectively, whereas for higher birth orders it extended upto 6 months. Especially for southern states, the MST of ANC service was found to be 3 months, however for EAG states including Assam, it was late as 5 months. Calculated Log-rank chi-square test value is significantly more than their tabulated value for respective degrees of freedom. For instance, tabulated χ^2^ value for degrees of freedom 2 at 5% level of significance for variable “age” is 5.99, whereas calculated value is 89.7, indicating that null hypothesis is rejected. Also *p* value is much less than 0.05 (< 0.05) which revealed that the survival functions of the categories for each characteristic under study were significantly different (Fig. 3).

### Predictors of the timing of the first ANC visit

All the study variables were incorporated in the Cox-PH model for regression analysis and adjusted for potential confounders by regression as described in Table 3. After adjustment age, educational level of women, residence, religion, wealth index, birth order, and region were found to be significant predictors of the timing of the first ANC. Keeping other factors constant, the timing of the first ANC visit was later (as per the standard recommendation) by 4% among women aged 25–34 (AHR: 0.96, CI:0.95–0.97) and 9% among women aged 35–49 (AHR:0.91, CI:0.90–0.93) as compared to 15–24 age group of women. Early initiation of ANC in pregnant women increased by 37% (AHR:1.37, CI:1.34–1.39) for primary education, 88% (AHR:1.88, CI:1.86–1.90) for secondary education and 181% (AHR:2.81, CI:2.76–2.86) for higher education compared to women having no formal education. Regarding the place of residence women in urban residents attained ANC of 49% (AHR:1.49, CI:1.47–1.51) higher compared to rural counterparts. Adjusting other factors initiation of ANC increased 1.50 times (AHR:1.50, CI:1.48–1.52) among women in the middle and increased 2.07 times (AHR:2.07, CI:2.05–2.10) in rich compared to poor wealth index. Holding other factors constant and assuming women in the same cluster, women with birth order 2 had a hazard of 27% (AHR:1.27, CI:1.25–1.28) and with birth order 3 had a hazard of 10% (AHR:1.10, CI:1.08–1.12) increased initiation of ANC compared to one parity. Similarly, women with birth order 4 and above had delayed initiation of ANC compared to parity 1 with a hazard ratio of 0.84 (AHR:0.84, CI:0.82–0.85).

**Table 3:** Multivariate Cox-proportional hazard model of potential predictors of timing of first antenatal care visit in India.

Variables	Category	Hazard ratio (95% CI)
Age	15–24	Reference
	25–34	0.96(0.95–0.97)**
	35–49	0.91(0.90–0.93)**
Education	No education	Reference
	Primary	1.37(1.34–1.39)**
	Secondary	1.88(1.86–1.90)**
	Higher	2.81(2.76–2.86)**
Residence	Rural	Reference
	Urban	1.49(1.47–1.51)**
Religion	Christian	Reference
	Hindu	1.18(1.16–1.21)**
	Muslim	1.05(1.02–1.07)**
	Others	1.45(1.40–1.49)*
Caste	Scheduled caste	Reference
	Scheduled tribe	0.95(0.93–0.96)
	Other backward classes	1.06(1.05–1.08)
	Others	1.35(1.33–1.37)*
Wealth index	Poor	Reference
	Middle	1.50(1.48–1.52)**
	Rich	2.07(2.05–2.10)**
Media expose	No	Reference
	Yes	1.56(1.52–1.60)
Birth order	1	Reference
	2	1.27(1.25–1.28)**
	3	1.10(1.08–1.12)**
	4 and above	0.84(0.82–0.85)**
Head of household	Female	Reference
	Male	1.03(1.01–1.04)
India’s region	Others	Reference
	Southern	1.00(0.98–1.02)**
	EAG + Assam	0.71(0.70–0.72)**

if ** 0.01; & * < 0.05 at the level of significance; EAG: Empowered Action Group.

## Discussion

Previous studies have talked a lot about the inequality in receiving ANC coverage within and between countries^13^, determinants of socio-economic inequality in maternal health care services such as ANC, institutional delivery and postnatal care^14–16^, multiple deprivations in utilizing maternal care services^17,18^, and examined the association between maternal care and health outcomes in India^5,19^. However, hardly any of the studies have tried to understand the socio-economic determinant and its estimates in mothers surviving by initiating the timing of the first ANC check-up in India as being home to one of the highest contributors to maternal mortality in the world^20^. Therefore this study provided an estimate of survival time using the initiation of time-to-first ANC visits among women in different socio-demographic groups in India. In fact, infant, child, and maternal mortality and morbidity remained the top global as well as a national priority in health development challenges, despite several international and national efforts have been made towards curbing the mortality rates and the multitude of resources directed to improving their surviving status. Improving maternal mortality and morbidity remained the top global as well as national priority despite several interventions, however present study is an effort to understand the utilization patterns of ANC services.

The study aimed to understand the time to first initiation of ANC and its predictors among pregnant women in India using National Family Health Survey data round four, 2015–2016^12^. Overall, it is found that at least one ANC checkup was assessed by 60.2 percent of women and the median survival time for the first ANC checkup among women was found to be 4 months. Furthermore, nearly one-quarter of pregnant women had booked ANC services within the first trimester at the national level, though it varied across different regions, for example, 5 months for EAG plus Assam states, 3 months for Southern states and 4 months for Others state. Most pregnant women had visited the first ANC check-ups after the first trimester which may have severe negative health repercussions in the later phase of pregnancy^3^. The reasons for the late initiation of the first ANC checkups among pregnant women have been influenced by many socio-economic and demographic factors. These factors have led to an increase the inequality in utilizing the first ANC services too. The predictor variables like age, caste, wealth quintile, maternal education, religion, birth order, place of residence, media exposure and the head of household are significantly associated with the time to the first initiation of ANC service among women. Exposure to mass media among women has also significantly influenced in utilizing the early ANC visits and initiated within the first trimester of their pregnancy. Our results have also coincided with other studies^16,21^. There are regional level variations in approaching first ANC services among women and found to be the north–south divide. Furthermore, the results also revealed how socio-economic and demographic factors operate in seeking the first ANC visit among women which is still highly prevailed in India.

The utilization of the first ANC visit is highly varied across regions of India. The recommended first initiation of ANC check-up was high in the southern region followed by the other region. The lowest is seen in the EAG plus Assam region. There could be several barriers in the EAG-plus Assam region including supply-side factors such as poor health infrastructure, shortages of human resources for health and other supply-side constraints that limit the utilizing the first ANC services in the first trimester^22^. The reasons might also include poor media exposure, knowledge, long distance to health facilities and lack of access to transportation^9,23^. Furthermore, the regional variation in the timing of the first ANC checkup in our study and its initiation has also been found significantly associated with the recent study by Goli et al., (2022) that the high prevalence and correlation of health care interventions and use of maternal care services to maternal mortality ratio across states and districts in India. There is a clear-cut bifurcation between the north–south divide in maternal health care utilization and maternal mortality ratio^19^. Similarly, our study has also provided evidence showing that northern states and regions are uneven and late in the initiation of the first ANC visits than the other regions which have direct implications on the survivorship of mothers and babies. They are more prone to a high risk for morbidity and mortality. The huge disparity in maternal mortality ratio has also been shown in accordance with the utilization patterns of ANC services^16,21^.

Our study has also highlighted the inequalities in the first ANC visits among women that were profound by the socio-demographic and economic groups at large. Earlier studies also show in line with this study that found the socio-economic determinants have still been prevalent and played a significant role in utilizing ANC services^14,15,17^. The result also revealed that the place of residence of the woman has affected the survival time by late initiation of the first ANC visit during the gestational age as coincided with the previous studies in Ethiopia and Nigeria respectively^8,9,11^.

Moreover, what is a more striking result of our study is that there is a huge socio-economic disparity in initiating time for the first ANC visit among pregnant women. Caste-associated inequality in receiving maternal health care services is huge in India^17,24^. The study clearly scrutinized the predictors that influenced the time to first ANC visit among pregnant women. There is a significant disparity in utilising the first ANC services. The results revealed that the lower caste women had taken a longer time and late initiated the first ANC check-up than the other caste groups. This late initiation of the first ANC services among the lower caste groups might result in high infant and maternal morbidity and mortality as earlier studies have clearly proven the phenomena^25^. At the same time, the association between household wealth status and time for the first ANC visit among women is found to be stronger. Women who belong to poor wealth quintiles received late and less ANC services than their counterparts^26–28^. Earlier studies have also demonstrated that economic inequality influences in receiving low maternal and child care services^17,29^. Despite the programme *Janani Suraksha Yojana* (JSY) introduced in India in 2005 under the National Rural Health Mission (NRHM) to provide monetary incentives to pregnant women and it is in place, however, still the out-of-pocket expenditure on maternal care is way higher than the usual incentives of JSY. Similarly, women’s education has also influenced on utilizing the first ANC visit. Low levels of education or illiterate women are less likely to start their first ANC check-up within the first trimester^23,30^. Our study has also confirmed in the line that illiterate women have lately initiated their first ANC visit as compared to their counterparts. Women's age and child birth order have significantly been associated with the early initiation of ANC visits. In fact, the increasing age of the mother has led to an increase in the timing of the first ANC visit, for instance, the age group of 35 and above clearly showed that their timing for the first ANC was 5 months. The high child-birth order (four & more) among women have increased the timing for the first ANC visit which was 5 months. These social determinants of women's health have influenced in the initiation of early ANC visits among pregnant women and increased the vulnerability in the timing for initiating the first ANC visit and that is after the first trimester.

There are pieces of evidence also that support the argument that by reducing high maternal and infant mortality rates, the timing for first antenatal care is one of the most pressing and determining factors and it is the essential component of maternal health care^11,22,25,28^. Therefore, early initiation and facilitation of ANC among pregnant women can determine pregnancy complications and save millions of mothers and babies^6^. The WHO has also recommended that pregnant women should begin early initiation to first ANC visits in the first trimester of their pregnancy. However, there are studies that reported that the MST is much higher in the countries like Ethiopia, Uganda^10^, and other developing countries too^31,32^. Our study also revealed that the median time of first ANC visits was 4 months which is delayed as per the WHO recommendations. Even it highly varied across regions and socio-economic groups. Across the major regions of India, the uptake of first ANC visits varied and the lowest is seen in southern states as compared to EAG plus Assam and other states. Poor socio-economic status- wealth quintile, caste, education, and religion; women’s demographic characteristics-age, sex, birth order, and head of the household; geographical locality- rural–urban, regions; and also exposure to mass media are found to be in a strong association.

There are kinds of literature that show the initiation of the first ANC visit in Nigeria is generally late and uneven with most of the women making their first ANC visit during the second trimester which is also highly varied across different socio-economic groups^7,11^. This late initiation of the first ANC visits among pregnant women has negative repercussions on their health as well as on babies' survivorship. Our results also revealed that most pregnant women in India have initiated their first ANC check-up late in the second trimester rather than in the first trimester. Therefore increasing necessary and routine antenatal care coverage among pregnant women may have several policy implications. This can ensure to all pregnant women the best health outcomes for their pregnancy and foetuses. The basic components of the ANC visits include risk identification, prevention and management of pregnancy-specific or concomitant diseases, education, and health promotion. This can evade emergency and obstetric mothers and newborn complications.

### Limitations

The study has some important strengths that it has used sample from a large nationally-representative survey NFHS-4 and to the best of our knowledge this study is the first of its kind in India to understand the socio-economic determinants that influence in early initiation of ANC visits and its estimation of survival time of pregnant women from pregnancy to childbirth. Despite this, we believe that there are some limitations to this study. Firstly, the survey did not collect information on why women initiated the first ANC late. Though there may be several reasons that could be responsible for the delay in starting the first ANC visit that may range from socio-economic and demographic factors to geographic, quality of care, perception and satisfaction of services. Furthermore, there can be a limitation as a recall bias among women. This is a cross-sectional study and can produce an association/correlation between ANC visits and socio-demographic factors. Unable to establish causality between outcomes variable and explanatory variables.

## Conclusion

The study concludes that the median survival time of the first ANC visit was 4 months which is highly delayed as compared to WHO recommendation of ANC visit among pregnant women in the country. Therefore, the study suggests that to prevent complications in pregnancy and childbirth, early detention is required. A high maternal, child, and infant mortality and morbidity can be averted through the proper interventions where to initiate early ANC visits among pregnant women. Furthermore, community health workers must be trained to provide health education to create community awareness regarding the timing of the first ANC visit earliest. Although the national health mission has put a concerted effort to improve the ANC coverage among pregnant women, the Accredited Social Health Activist (ASHA) programme has played a crucial role in motivating and behavioural change for utilizing more ANC services. However, there is still a huge inequality in receiving first ANC services across different socio-economic groups.

## Data Availability

The study utilises secondary source of data which is freely available in public domain through http://rchiips.org/nfhs/data1.shtml.

## Abbreviations

NFHS: National Family Health Survey
MMR: Maternal Mortality Ratio
SRS: Sample Registration System
MOHFW: Ministry of Health and Family Welfare
UNICEF: United Nations Children’s Fund
WHO: World Health Organization
NRHM: National rural health mission
PSU: Primary sampling unit
CEB: Census enumeration block
ANC: Antenatal care
MST: Median survival time
KM test: Kaplan–Meier test
Cox PH: Cox proportional hazard
AHR: Adjusted hazard ratio
CI: Confidence interval
